# Comparative analysis of the effect of electromyogram to bispectral index and 95% spectral edge frequency under remimazolam and propofol anesthesia: a prospective, randomized, controlled clinical trial

**DOI:** 10.3389/fmed.2023.1128030

**Published:** 2023-08-07

**Authors:** Yueyang Xin, Li Ma, Tianli Xie, Yuhui Liang, Miao Ma, Tiantian Chu, Cheng Liu, Aijun Xu

**Affiliations:** ^1^Department of Anesthesiology, Hubei Key Laboratory of Geriatric Anesthesia and Perioperative Brain Health, Wuhan Clinical Research Center for Geriatric Anesthesia, Tongji Hospital, Tongji Medical College, Huazhong University of Science and Technology, Wuhan, Hubei, China; ^2^School of Information Engineering, Wuhan University of Technology, Wuhan, Hubei, China

**Keywords:** correlation, sedation, remimazolam, bispectral index, electromyogram, 95% spectral edge frequency

## Abstract

**Background:**

Bispectral index (BIS), an index used to monitor the depth of anesthesia, can be interfered with by the electromyogram (EMG) signal. The 95% spectral edge frequency (SEF95) also can reflect the sedation depth. Remimazolam in monitored anesthesia care results in higher BIS values than propofol, though in the same sedation level assessed by Modified Observers Assessment of Alertness and Sedation (MOAA/S). Our study aims to illustrate whether EMG is involved in remimazolam causing higher BIS value than propofol preliminarily and to explore the correlations among BIS, EMG, and SEF95 under propofol and remimazolam anesthesia.

**Patients and methods:**

Twenty-eight patients were randomly divided into propofol (P) and remimazolam (RM) groups. Patients in the two groups received alfentanil 10 μg/kg, followed by propofol 2 mg/kg and remimazolam 0.15 mg/kg. Blood pressure (BP), heart rate (HR), and oxygen saturation (SpO_2_) were routinely monitored. The BIS, EMG, and SEF95 were obtained through BIS VISTATM. The primary outcomes were BIS, EMG, and the correlation between BIS and EMG in both groups. Other outcomes were SEF95, the correlation between BIS and SEF95, and the correlation between EMG and SEF95. And all the statistical and comparative analysis between these signals was conducted with SPSS 26.0 and GraphPad Prism 8.

**Results:**

BIS values, EMG, and SEF95 were significantly higher in the RM group than in the P group (all *p* < 0.001). There was a strong positive correlation between BIS and EMG in the RM group (*r* = 0.416). Nevertheless, the BIS in the P group showed a weak negative correlation with EMG (*r* = −0.219). Both P (*r* = 0.787) and RM group (*r* = 0.559) had a reasonably significant correlation coefficient between BIS and SEF95. SEF95 almost did not correlate with EMG in the RM group (*r* = 0.101).

**Conclusion:**

Bispectral index can be interfered with high EMG intensity under remimazolam anesthesia. However, EMG can hardly affect the accuracy of BIS under propofol anesthesia due to low EMG intensity and a weak negative correlation between EMG and BIS. Moreover, SEF95 may have a great application prospect in predicting the sedation condition of remimazolam.

## 1. Introduction

Monitoring anesthesia depth during surgery is vital in preventing perioperative complications such as body movement and delayed recovery (1). Electroencephalogram (EEG) changes are the gold standard for determining the depth of anesthesia (2). EEG monitoring has been used in clinical anesthesia, and EEG-based monitoring techniques guide anesthesia management (3, 4). The bispectral index (BIS) is a dimensionless number constant in the 0–100 range. It calculates from four parameters in EEG: Relative BetaRatio, QUAZI suppression, SynchFastSlow, and Burst Suppression (5). Electromyogram (EMG) signal higher than 30–40 Hz due to facial muscle activity can cause bias in BIS values (6). Because the signal spectrum range produced by EMG overlaps precisely with the 30–47 Hz range in which Relative BetaRatio is needed to calculate the BIS value; the EMG signal will affect the accuracy of the BIS value (7). Studies showed that for intensive care unit (ICU) patients requiring sedation of propofol (8), midazolam (9), or isoflurane (9) without neuromuscular blocker, the enhancement of EMG signal significantly correlates with increased BIS value, which will bring confusion to the judgment on the depth of sedation. Hence, when BIS is used to monitor the depth of sedation, the influence of EMG activity must be considered to avoid misestimation of the depth of hypnosis, resulting in sedated drug overdose or insufficiency.

95% spectral edge frequency (SEF95) is calculated from the sinusoidal component of the EEG power spectrum after Fourier transforms and reflects the frequency threshold below which 95% of the total signal power is contained (10, 11). In awake subjects, the central frequency of EEG is beta rhythm (>13 Hz) (12). Under general anesthesia, it is characterized and dominant by slow waves in the delta-band frequency (<4 Hz) and alpha-band (8–12 Hz) activities (13). Morimoto et al. showed that when the BIS value was 30–80, SEF95 had an excellent correlation with BIS (14). Moreover, SEF95 also has the potential to assess the depth of anesthesia during surgery (15).

Remimazolam, a novel short-acting intravenous benzodiazepine, acts as a positive allosteric modulator of γ-aminobutyric acid subtype A (GABA_A_) receptor *via* benzodiazepine binding site (16, 17). Compared with propofol, remimazolam has a higher BIS under general anesthesia (18–20). Similarly, our previous study found that 0.15 mg/kg remimazolam can achieve the same sedation level assessed by MOAA/S as 2 mg/kg propofol did. In the meantime, patients in the remimazolam group had higher BIS values than those in the propofol group (21). Accordingly, there is still no conclusion about whether EMG is involved in higher BIS during monitored anesthesia care with remimazolam. Therefore, our study is to evaluate whether EMG involved in remimazolam causes higher BIS value than propofol preliminarily and to explore the correlations among BIS, EMG, and SEF95 under propofol and remimazolam anesthesia.

## 2. Materials and methods

### 2.1. Study design and patients

This prospective, randomized, controlled pilot trial was planned to observe patients undergoing colonoscopic polypectomy in Tongji Hospital. The trial was registered before patient enrollment at http://www.chictr.org.cn (principal investigator: Aijun Xu, date of registration and registration number: 05/08/2022, ChiCTR2200062413) Tongji Medical College of Huazhong University of Science and Technology Ethics Committee (IORG No: IORG0003571) approved the trial’s conduction. Written informed consent was obtained from all subjects participating in our trial. This trial follows applicable Consolidated Standards of Reporting Trials (CONSORT) guidelines.

Patients who received the colonoscopic polypectomy in Tongji Hospital from August 6 to September 5, 2022. They were evaluated following the inclusion criteria: (1) aged 18–80 years; (2) American Society of Anesthesiologists (ASA) status I or II; (3) Body Mass Index (BMI) 18.5–23.9 kg/m^2^; (4) operation time is 20–60 min. The exclusion criteria as shown below: (1) emergency operation; (2) allergic to benzodiazepines and opioids; (3) high risk of a full stomach and reflux aspiration; (4) taking the analgesic, sedative, or antidepressant drugs within 24 h; (5) pregnant or breastfeeding; (6) renal or liver dysfunction; (7) drug abuse; (8) participated in other clinical studies recently; (9) cannot cooperate or communicate. Investigators determined that the patient withdrew using the following criteria: poor compliance or severe complications, such as postoperative intestinal perforation needing emergency surgery and severe infection. Detailed reasons will be recorded in the case report form (CRF) for reference.

### 2.2. Randomization and grouping

Participants were randomly assigned to receive either remimazolam or propofol induction. According to the randomized number generated through the Statistical Package for Social Sciences (SPSS) software version 26.0, patients were randomized into the remimazolam or propofol group. Researchers who were not involved in anesthesia management implemented randomization. Randomized numbers were sealed in numbered opaque envelopes. In this trial, we adopted a single-blind study method. An anesthesiologist with more than 10 years of working experience performed preoperative evaluation, anesthesia management, and intraoperative data collection. Finally, after all the enrolled patients’ data were collected, the opaque envelopes only were opened by the good clinical practice (GCP) monitor and investigators. Statistical experts from Tongji Hospital and Wuhan University of Technology analyzed the final data. All researchers except anesthesiologists were blinded to the grouping.

### 2.3. Anesthesia induction and maintenance

Patients underwent bowel preparation on the day before their surgery. They were established venous access and introduced 250 ml 0.9% sodium chloride solution after they were brought to the endoscopy room. An oxygen inhalation mask was administered immediately at a rate of 3 L/min. Blood pressure (BP), heart rate (HR), and oxygen saturation (SpO_2_) were routinely monitored. The BIS, EMG, and SEF95 were obtained through BIS VISTA™ (Aspect Medical Systems, Inc., Norwood, U.S.A.). The patients in the propofol (P) group were administered propofol (Corden Pharma S.P.A., RX061) 2 mg/kg (22–24) and alfentanil (Yichang Humanwell Pharmaceutical, Co., Ltd., China, 13S03051) 10 μg/kg. Patients in the remimazolam (RM) group received remimazolam besylate (Yichang Humanwell Pharmaceutical, Co., Ltd., China, 70,705,021) at 0.15 mg/kg and alfentanil 10 μg/kg. It took over 1 min to induce sedation for all patients. Colonoscopic polypectomy was performed by the same endoscopist with over 10 years of experience. An additional 1/3 to 1/2 of the initial dose of propofol or alfentanil and 2.5 mg remimazolam were administered to keep the appropriate sedation (MOAA/S ≤ 1) and painless. If hypotension (20% lower than baseline value) and bradycardia (HR is less than 60 beats per minute) occur, ephedrine and atropine were given to maintain circulation stability. We used artificial assisted ventilation when SpO_2_ decreased to less than 90% and sustained for more than 20 s, regarded as respiratory depression associated with sedation (25).

### 2.4. Outcome measures

The primary outcomes were BIS, EMG, and the correlation between BIS and EMG in both groups. Other outcomes were SEF95, the correlation between BIS and SEF95, and the correlation between EMG and SEF95. Data were extracted from BIS VISTA™ (Aspect Medical Systems, Inc., Norwood, U.S.A.) and were extracted per second during the whole colonoscopic polypectomy for following analysis. The EMG exported by BIS VISTA™ sums the spectral power between 70 and 110 Hz and is defined as the power in decibels (dB). Baseline demographics and case characteristics were recorded, such as age, sex, BMI, mean artery pressure (MAP), HR, SpO_2_, ASA classification, BIS, EMG, and SEF95 before anesthesia.

### 2.5. Statistical analysis and sample size calculation

SPSS software version 26.0 (SPSS Inc., Chicago, IL, USA) and GraphPad Prism 8 (GraphPad Software, San Diego, CA, USA) were used for statistical analysis. The Kolmogorov–Smirnov test was used to determine the normal distribution of continuous variables. The continuous variables were expressed as the mean ± standard deviation (SD) or median (interquartile range). Categorical data were expressed as the number (percentage). Continuous variables were analyzed using the Mann–Whitney *U* test and independent-samples *T* test or Welch *T* test based on the homogeneity of variance test. Categorical variables were compared using Pearson’s chi-square test or Fisher’s exact test. We used scatter plots and did a linear regression analysis using the least squares method. We plotted the linear regression line and performed a one-sided F-test to compare the slope coefficient against zero. We applied Spearman’s correlation analysis to detect the correlation among BIS, EMG, and SEF95. The *p* value <0.05 was considered statistically significant. We used Cohen’s d to indicate the effect size. Furthermore, we explored intervention effects within the specific subgroup, that is, gender (female, male).

We used the difference in BIS values based on our previous clinical trials (21) to calculate the sample size. We utilized Power Analysis and Sample Size (PASS) 15.0.5 software to calculate based on the following parameters: BIS value for propofol (58.7 ± 3.5) and remimazolam (64.6 ± 2.3), the ratio of remimazolam group to propofol group was 1:1, power = 0.95, *α* = 0.025. A sample size of 10 per group was calculated. To account for the incomplete data recording, we decided to include 28 patients, 14 cases for each group in this study.

## 3. Results

**Figure 1:**
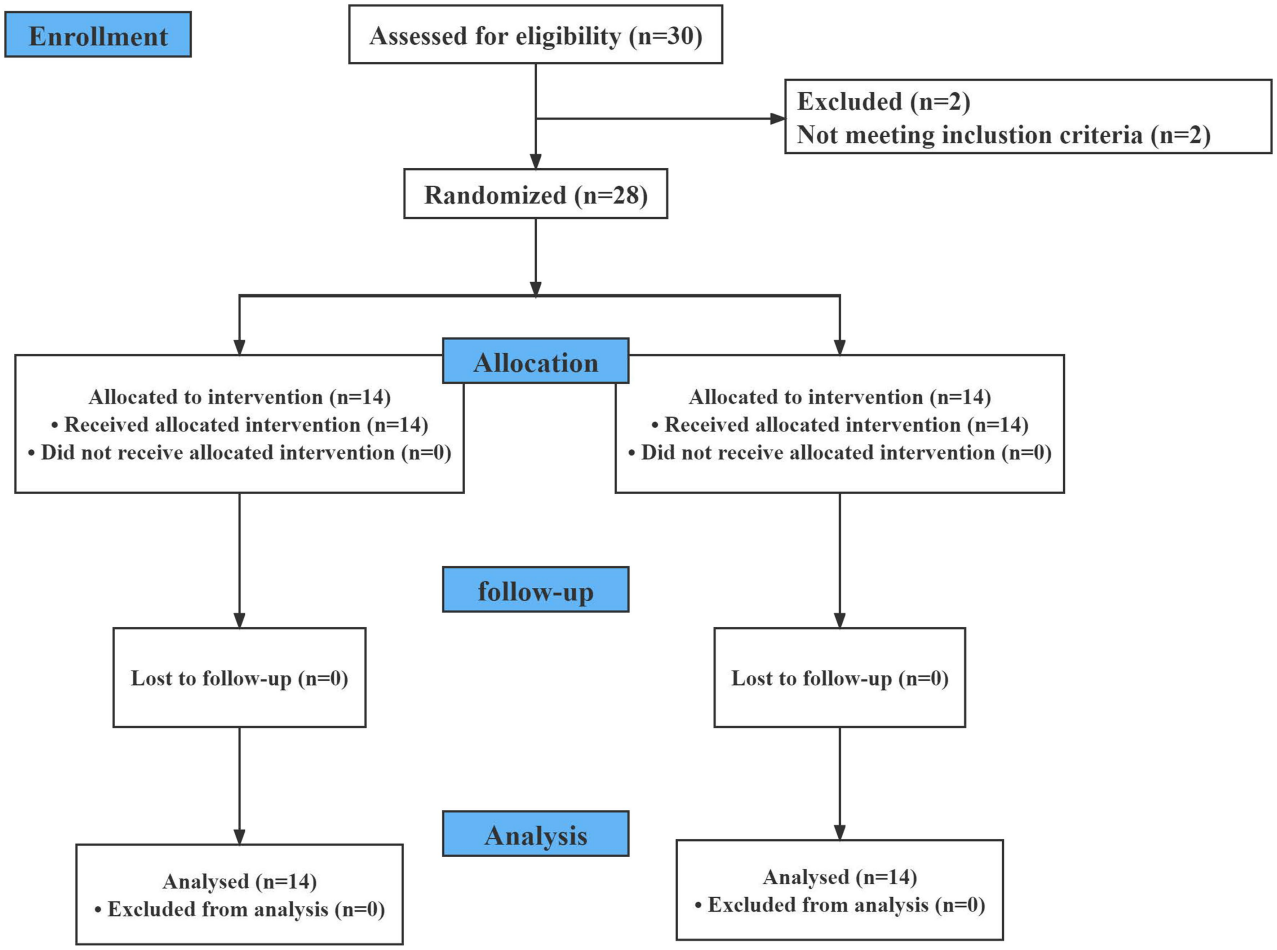
CONSORT flow diagram of participants.

Thirty patients were assessed for eligibility, and two were excluded because of BMI and cerebral infarction. Twenty-eight patients were randomized into two groups (*n* = 14 for each group, Figure 1). The baseline demographic was presented in Table 1.

**Table 1:** Patient demographics.

Characteristics	P group (*n* = 14)	RM group (*n* = 14)	*p* values
Age, years	47.36 ± 14.35	53.64 ± 11.44	*p* = 0.256
Male/female	8/6	9/5	*p* = 0.699
BMI, kg/m^2^	21.66 ± 2.02	22.12 ± 1.81	*p* = 0.477
Mean artery pressure, mmHg	90.86 ± 5.36	89.29 ± 8.18	*p* = 0.144
HR, bpm	77.43 ± 6.96	78.64 ± 7.45	*p* = 0.524
SpO_2_, %	98.00 (97.75, 99.00)	98.00 (98.00, 99.00)	*p* = 0.125
BIS	96.65 (94.08, 97.50)	96.70 (94.40, 97.63)	*p* = 0.804
EMG	47.69 ± 3.35	47.74 ± 4.85	*p* = 0.979
SEF95	23.06 ± 1.87	23.65 ± 2.08	*p* = 0.435
ASA classification			
I	11 (78.6%)	10 (71.4%)	*p* = 0.705
II	3 (21.4%)	4 (28.6%)	

Data are presented as number, mean ± SD, median (interquartile range), and number (percentage). P, propofol; RM, remimazolam; HR, heart rate; SpO_2_, oxygen saturation; BIS, bispectral index, EMG, electromyogram, SEF95, 95% spectral edge frequency, ASA, American Society of Anesthesiologists, BMI, body mass index.

### 3.1. Primary outcomes

Each patient has randomly selected 300 sampling points from the maintenance phase of the colonoscopic polypectomy. All the sampling points’ signal quality index (SQI) was all above 90 and we used 300 sampling points to conduct subsequent data analysis. The BIS value was significantly higher in the RM group (*p* < 0.001, Table 2) than in the P group. Similarly, in the RM group, EMG (*p* < 0.001, Table 2) and SEF95 (*p* < 0.001, Table 2) were both significantly higher than the P group. All three variables had a significant effect size.

**Table 2:** Comparison of BIS, EMG, and SEF95 between the P group and the RM group.

	P group (*n* = 14)	RM group (*n* = 14)	Cohen’s *d*	*P-*values
BIS	59.73 ± 7.02	69.79 ± 6.30	1.508	*p* < 0.001
EMG (dB)	29.48 ± 2.70	33.53 ± 5.68	0.911	*p* < 0.001
SEF95	18.60 ± 2.36	21.34 ± 1.72	1.327	*p* < 0.001

Data are presented as mean ± SD and number. P, propofol; RM, remimazolam; BIS, bispectral index; SEF95, 95% spectral edge frequency; EMG, electromyogram.

As for the correlation between BIS and EMG, our results showed that BIS in the P group (*r* = −0.219, Table 3) had a weak negative correlation with EMG. Nevertheless, BIS in the RM group (r = 0.416, Table 4) had a moderate positive correlation with EMG, which indicated that EMG contributes significantly to BIS values in remimazolam sedated rather than propofol.

**Table 3:** Comparison between EMG and BIS vs. EMG and SEF95 under propofol anesthesia.

	Spearman’s correlation coefficient and 95% CI	Linear model	95% CI of the slope	*F*-statistics	*P-v*alues	*R*^2^
BIS	−0.279 [−0.306, −0.252]	BIS = -0.592*EMG + 77.21	[−0.669, −0.516]	231.0	*p* < 0.0001	0.052
SEF95	−0.471 [−0.492, −0.449]	SEF95 = -0.379*EMG + 29.79	[−0.401, −0.355]	979.9	*p* < 0.0001	0.189

Data are presented as number, 95% confidence interval, and number (95% confidence interval). BIS, bispectral index; CI, confidence interval; EMG, electromyogram; SEF95, 95% spectral edge frequency.

**Table 4:** Comparison between EMG and BIS vs. EMG and SEF95 under remimazolam anesthesia.

	Spearman’s correlation coefficient and 95% CI	Linear model	95% CI of the slope	*F*-statistics	*P-v*alues	*R*^2^
BIS	0.416 [0.388, 0.444]	BIS = 0.474*EMG + 53.90	[0.444, 0.504]	935.3	*p* < 0.0001	0.182
SEF95	0.101 [0.069, 0.131]	SEF95 = 0.013*EMG + 20.91	[0.004, 0.022]	7.474	*p =* 0.0063	0.002

Data are presented as number, 95% confidence interval, and number (95% confidence interval). BIS, bispectral index; CI, confidence interval; EMG, electromyogram; SEF95, 95% spectral edge frequency.

**Figure 2:**
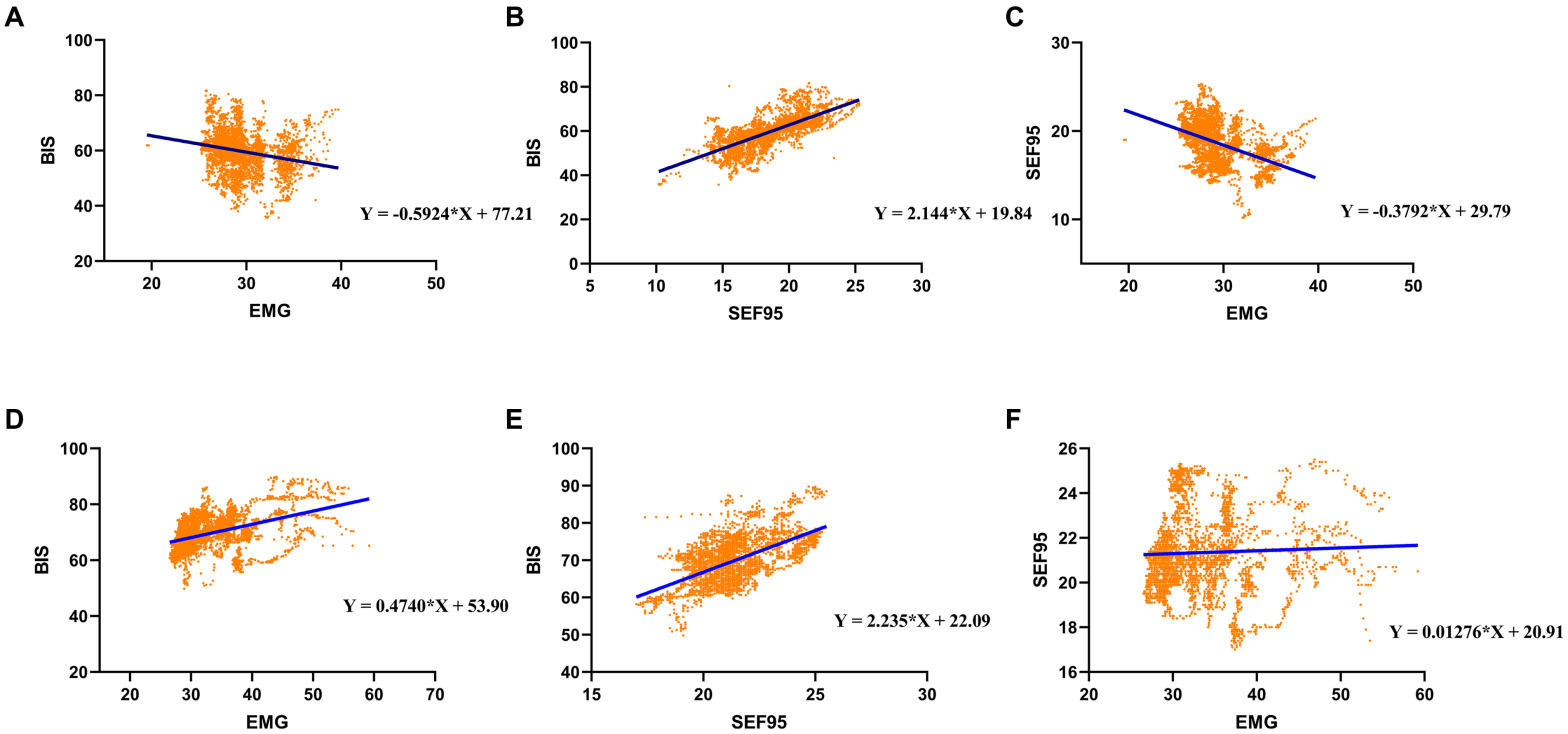
Linear regression for **(A)** BIS and EMG, **(B)** BIS and SEF95, and **(C)** SEF95 and EMG under propofol anesthesia; Linear regression for **(D)** BIS and EMG, **(E)** BIS and SEF95, and **(F)** SEF95 and EMG under remimazolam anesthesia.

For the P group, BIS significantly decreased with EMG by around 0.59 index points per EMG (Figure 2A and Table 3). The BIS significantly increased with EMG by approximately 0.47 index points per dB (Figure 2D and Table 4) under remimazolam anesthesia.

### 3.2. Other outcomes

As for the SEF95 and EMG, our results showed that SEF95 in the P group (*r* = −0.471, Table 3) showed a moderate negative correlation with EMG. However, in the RM group, SEF95 seldom correlates with EMG (*r* = 0.101, Table 4). Besides, The P group had a strong correlation between the BIS and SEF95 (*r* = 0.787, Table 5). Similarly, the RM group had a moderate correlation between BIS and SEF95 (r = 0.559, Table 5).

**Table 5:** Comparison between SEF95 and BIS under propofol and remimazolam anesthesia.

	Spearman’s correlation coefficient and 95% CI	Linear model	95% CI of the slope	*F*-statistics	*P-v*alues	*R*^2^
BIS (P group)	0.787 [0.774, 0.800]	BIS = 2.144*SEF95 + 19.84	[2.082, 2.207]	4,531	*p* < 0.0001	0.519
BIS (RM group)	0.559 [0.537, 0.581]	BIS = 2.235*SEF95 + 22.09	[2.147, 2.323]	2,479	*p* < 0.0001	0.371

Data are presented as number, 95% confidence interval, and number (95% confidence interval). P, propofol; RM, remimazolam; BIS, bispectral index; CI, confidence interval; SEF95, 95% spectral edge frequency.

For the P group, SEF95 significantly decreased with EMG by around 0.38 index points per EMG (Figure 2C and Table 3). However, in the RM group, SEF95 was unaffected by EMG (Figure 2F and Table 4), which was different compared with the P group. Both in the P group and the RM group, BIS significantly increased with SEF95 by around 2.2 index points per SEF95 (Figures 2B,E and Table 5).

Exploratory subgroup analysis concerning gender showed similar results between the P group and the RM group in the primary outcomes and other outcomes as above (Supplementary Figures S1, S2 and Supplementary Tables S1–S8).

In order to explain why there was a negative correlation between BIS and EMG in the P group, sensitivity analysis was conducted. Due to the result that EMG was higher in the RM group, we hypothesized that only EMG higher than a “threshold value” can falsely elevated the BIS value. Therefore, we divided preliminarily according to the mean value of EMG. When EMG was less than 29.5 dB, we still found a significant negative correlation between BIS and EMG. BIS decreased with EMG by 1.055 index points per EMG, which is statistically significant (Table 6). When EMG was higher than or equal to 29.5 dB, six sub-groups were partitioned at 0.5 dB. However, only EMG higher than or equal to 31.5 and 32 dB indicated a significant positive correlation between BIS and EMG. BIS significantly increased with EMG by around 1.030 and 2.173 index points per dB, respectively (Table 6). This was contrary to the previously found correlation between BIS and EMG. Moreover, *R*^2^ was bigger when EMG was higher than or equal to 32 dB.

**Table 6:** Comparison between EMG and BIS at different EMG levels under propofol anesthesia.

EMG level (dB)	Spearman’s correlation coefficient and 95% CI	Linear model	95% CI of the slope	*F*-statistics	*P-v*alues	*R*^2^
<29.5	−0.128 [−0.167, −0.087]	BIS = -1.055*EMG + 90.38	[−1.293, −0.816]	75.16	*p* < 0.0001	0.027
≥29.5	−0.011 [−0.062, 0.036]	NA	NA	NA	NA	NA
≥30	0.034 [−0.018, 0.091]	NA	NA	NA	NA	NA
≥30.5	0.012 [−0.052, 0.076]	NA	NA	NA	NA	NA
≥31	−0.002 [−0.070, 0.064]	NA	NA	NA	NA	NA
≥31.5	0.175 [0.106, 0.248]	BIS = 1.030*EMG + 21.78	[0.703, 1.358]	38.13	*p* < 0.0001	0.047
≥32	0.287 [0.213, 0.361]	BIS = 2.173*EMG-18.32	[1.770, 2.577]	111.6	*p* < 0.0001	0.138

Data are presented as number, 95% confidence interval, and number (95% confidence interval). BIS, bispectral index; CI, confidence interval; EMG, electromyogram; NA, not applicable.

## 4. Discussion

Current theories indicate that anesthetics induce unconsciousness by acting on different brain regions (26). The brain’s electrical activity, known as EEG, can be recorded *via* forehead electrodes (4). Thus, we quantify the effects on the brain using EEG analysis during anesthesia (26). However, the interpretation of raw EEG is time-consuming and intricate (27). With the help of depth-of-anesthesia monitors, we utilize processed EEG signals to represent the depth of anesthesia and guide decisions (28). The BIS and SEF95 are two applied parameters of processed EEG (4). Besides, EMG data are often incorporated into algorithms of processed EEG; it is usually isolated for separate display from depth-of-anesthesia index (28). Accordingly, the BIS, EMG, and SEF95 are critical in indicating the depth of anesthesia in the operation room.

Bispectral index monitoring, a method used to assess the depth of anesthesia, may reduce the risk of intraoperative awareness and maintain an accurate depth of anesthesia, which helps in early postoperative recovery (29). Our research found that BIS values were significantly higher in the RM group during operation. The mean BIS values in the P and RM groups were 59.73 and 69.79, respectively. It is similar to previous studies (18–21). Moreover, for the RM group, the intensity of EMG was also higher than the P group. However, EMG activity can significantly influence BIS monitoring and mislead the anesthetist to re-adjust the depth of anesthesia (30, 31). Studies demonstrated that EMG_30-150 Hz_ overlapped EEG_30-47 Hz_, which was correlated to the BetaRatio, and the signal intensity of EMG was more extensive than that of EEG. Therefore, the EMG_30–150 Hz_ portion could interfere with BetaRatio and BIS calculation (32, 33). Several studies showed a significant increase in BIS values when patients were administered muscle relaxant antagonists (34–36). Moreover, the rise of BIS was accompanied by an increase in EMG (34–36). Similarly, BIS has positively correlated with EMG activity in various conditions: coronary artery bypass graft surgery (37), propofol and sufentanil for sedation (8), and combined anesthesia (30). Our study indicated that BIS and EMG values had a positive correlation in patients who received remimazolam for colonoscopic polypectomy, which was in line with the above studies.

However, Shirozu et al. indicated that EMG did not correlate with BIS during remimazolam anesthesia (20, 38). This is probably due to the use of rocuronium, which inevitably affects the intensity of EMG. Besides, BIS data greater than 60 were not included in the analysis. Experimental design and data analysis methods might also affect study results to some extent. There was a weak negative correlation between EMG and BIS for patients who received propofol in our study, contrary to the previous conclusion (8, 39). The possible reasons are described below: First, anesthetics used in different studies varied (sufentanil-propofol vs. remifentanil-propofol vs. alfentanil-propofol). Second, in the other two studies, the EMG signal intensity included in the analysis was above 35 dB. In our research, the intensity of EMG is mostly below 35 dB. Further sensitivity analysis for the P group in our study showed that a positive correlation existed only when EMG was equal to or greater than 31.5 dB. The analysis included no correlation or a weak negative correlation between BIS and EMG when a smaller intensity of the EMG signals was included. We may infer that EMG with smaller signal intensity may have limited or no interference with BIS. There is a “threshold value” for EMG to falsely increase the BIS values. Third, differences in monitoring equipment and population should be considered. In the meantime, a few studies suggested that the decrease in EMG intensity due to muscle relaxants did not affect BIS values in patients who received propofol anesthesia (40, 41). Therefore, more research is still needed to illuminate whether and how EMG affects BIS in different anesthetics.

SEF95, one of the processed EEG indices, could also be used to predict sedation levels but exhibited large interindividual variability (42). Morimoto et al. showed that the BIS had a strong positive correlation with SEF95 during isoflurane anesthesia (14). Similarly, our study indicated that there was also a significant positive correlation between BIS and SEF95 for patients who received either propofol anesthesia or remimazolam anesthesia. In the P group, a moderate negative correlation existed between EMG and SEF95. Moreover, there was almost no correlation between EMG and SEF95 in the RM group. No research has focused on the correlation between EMG and SEF95 before. The most plausible explanation of our results is that remimazolam resulted in higher EMG and SEF95 than propofol; there may be a different correlation between EMG and SEF95 at different levels. We have demonstrated that EMG influenced BIS during remimazolam anesthesia. Thus, we hypothesize that SEF95 may be more efficient in predicting sedation levels in patients who received remimazolam anesthesia. More prospective clinical studies are needed to confirm our hypothesis.

Although in this trial, gender did not affect the primary outcomes and other outcomes, it is reported that women had higher BIS values at similar concentrations of anesthesia (43). Meanwhile, Gross et al. suggested that women were associated with increased periods of BIS <40 during the maintenance of total intravenous anesthesia (44). These results may be related to the influence of gender factors on the metabolism of anesthetics such as propofol (45, 46). Therefore, the possible influence of gender should be considered in the future study of BIS or the depth of anesthesia.

This study has the following limitations: First, we only focused on the relationship among BIS, EMG, and SEF95 during the maintenance period. The relationship among the three parameters in the induction and recovery periods still needs to be elucidated. Second, it was a single-center study, our findings may not possess universality. Further studies are needed to validate the present results and aim to elucidate more elaborately.

In conclusion, BIS in patients who received remimazolam for monitored anesthesia care can be interfered with the high EMG intensity and had a moderate positive correlation with EMG. However, EMG can hardly affect the accuracy of BIS under propofol anesthesia probably because the propofol resulted in a low EMG intensity and a weak negative correlation between EMG and BIS. Although there was a positive correlation between BIS and SEF95 in both the remimazolam group and propofol group, SEF95 may have a great application prospect in predicting the sedation condition of remimazolam because of almost no correlation with EMG intensity.

## Data availability statement

The original contributions presented in the study are included in the article/Supplementary material, further inquiries can be directed to the corresponding author.

## Ethics statement

The studies involving human participants were reviewed and approved by Tongji Medical College of Huazhong University of Science and Technology Ethics Committee. The patients/participants provided their written informed consent to participate in this study.

## Author contributions

YX: conceptualization, data curation, formal analysis, investigation, and writing – original draft. LM: conceptualization, data curation, methodology, software, writing – review and editing, and funding acquisition. TX: data curation, formal analysis, and software. YL: data curation, formal analysis, and software. MM: formal analysis and software. TC: investigation and writing – original draft. CL: conceptualization and writing – review and editing. AX: conceptualization, project administration, resources, supervision, and writing – review and editing. All authors read and approved the final manuscript.

## Funding

This research was supported in part by the financial support of the Natural Science Foundation of Hubei Province, grant no. 2022CFB896.

## Conflict of interest

The authors declare that the research was conducted in the absence of any commercial or financial relationships that could be construed as a potential conflict of interest.

## Publisher’s note

All claims expressed in this article are solely those of the authors and do not necessarily represent those of their affiliated organizations, or those of the publisher, the editors and the reviewers. Any product that may be evaluated in this article, or claim that may be made by its manufacturer, is not guaranteed or endorsed by the publisher.

## Abbreviations

ANOVA: analysis of variance
ASA: American Society of Anesthesiologists
BIS: bispectral index
BMI: Body Mass Index
BP: blood pressure
CI: confidence interval
CONSORT: Consolidated Standards of Reporting Trials
CRF: case report form
dB: decibels
GABA_A_: γ-aminobutyric acid subtype A
GCP: good clinical practice
EEG: electroencephalogram
EMG: electromyogram
HR: heart rate
ICU: intensive care unit
MAP: mean arterial pressure
MOAA/S: Modified Observers Assessment of Alertness and Sedation
NA: not applicable
P: propofol
PASS: Power Analysis and Sample Size
RM: remimazolam
SD: standard deviation
SEF95: 95% spectral edge frequency
SPSS: Statistical Package for Social Sciences
SpO_2_: oxygen saturation
SQI: signal quality index
