# Supplementary material for: Comparative analysis of the effect of electromyogram to bispectral index and 95% spectral edge frequency under remimazolam and propofol anesthesia: a prospective, randomized, controlled clinical trial
Source: Front Med (Lausanne). 2023 Aug 7;10:1128030. doi: 10.3389/fmed.2023.1128030 (PMC10442164; doi:10.3389/fmed.2023.1128030)
Supplement: Supplementary file 1 [file Data_Sheet_1.docx]

Supplementary Material

Comparative analysis of the effect of electromyogram to bispectral index and 95% spectral edge frequency under remimazolam and propofol anesthesia: a prospective, randomized, controlled clinical trial

Yueyang Xin^1†^, Li Ma^2†^, Tianli Xie^2^, Yuhui Liang^2^, Miao Ma^2^, Tiantian Chu^1^, Cheng Liu^1^, Aijun Xu ^1*^

^1^ Department of Anesthesiology, Hubei Key Laboratory of Geriatric Anesthesia and Perioperative Brain Health, and Wuhan Clinical Research Center for Geriatric Anesthesia, Tongji Hospital, Tongji Medical College, Huazhong University of Science and Technology

^2^ School of Information Engineering, Wuhan University of Technology, 122 Luoshi Road, Wuhan, Hubei 430070 China

**† These authors contributed equally to this work and shared the first authorship**

*** Correspondence:**Aijun Xu
ajxu@tjh.tjmu.edu.cn

# Supplementary Figures and Tables

## Supplementary Figures

**
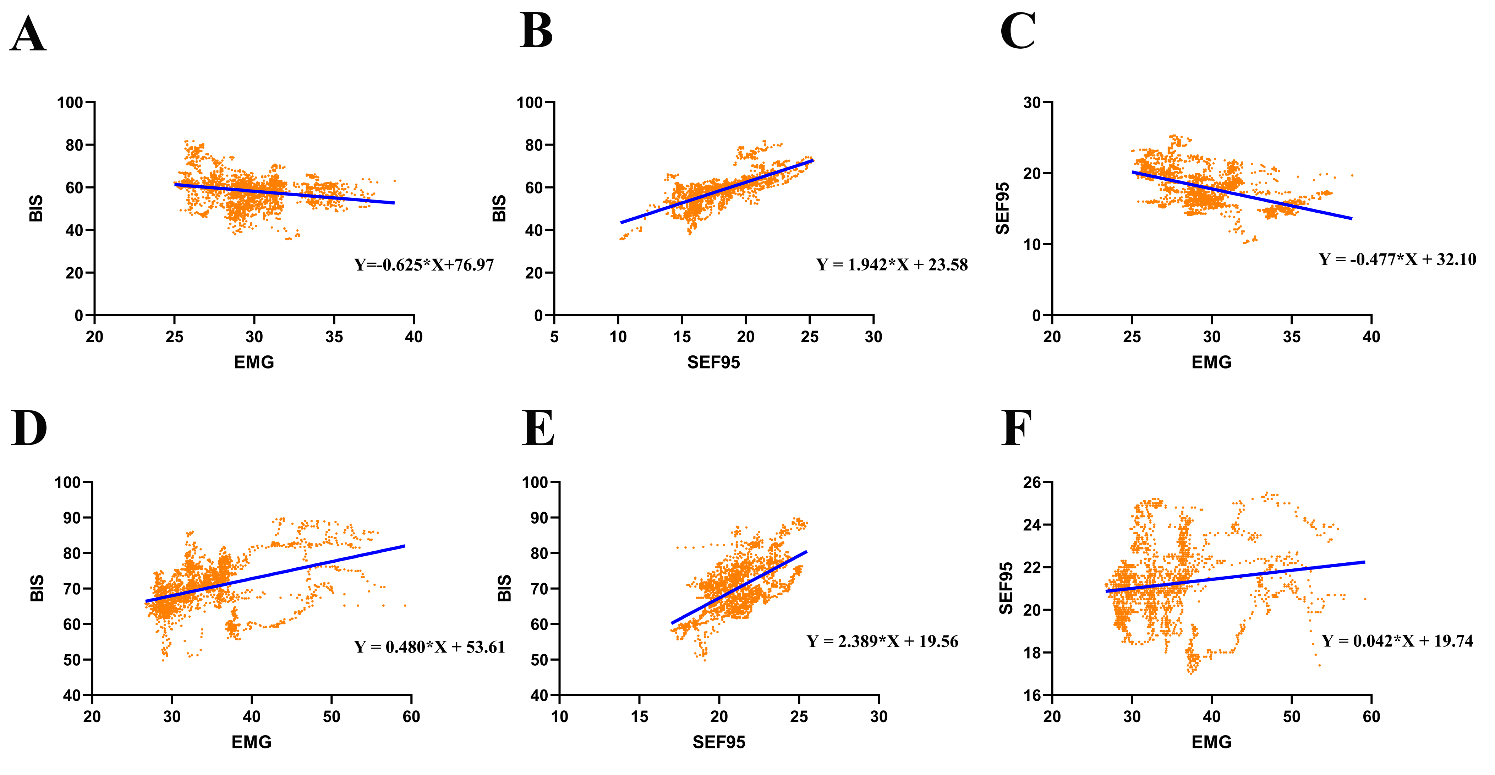
**

**Supplementary Figure 1.** Linear regression for A: BIS and EMG, B: BIS and SEF95, and C: SEF95 and EMG under propofol anesthesia in males; Linear regression for D: BIS and EMG, E: BIS and SEF95, and F: SEF95 and EMG under remimazolam anesthesia in males.


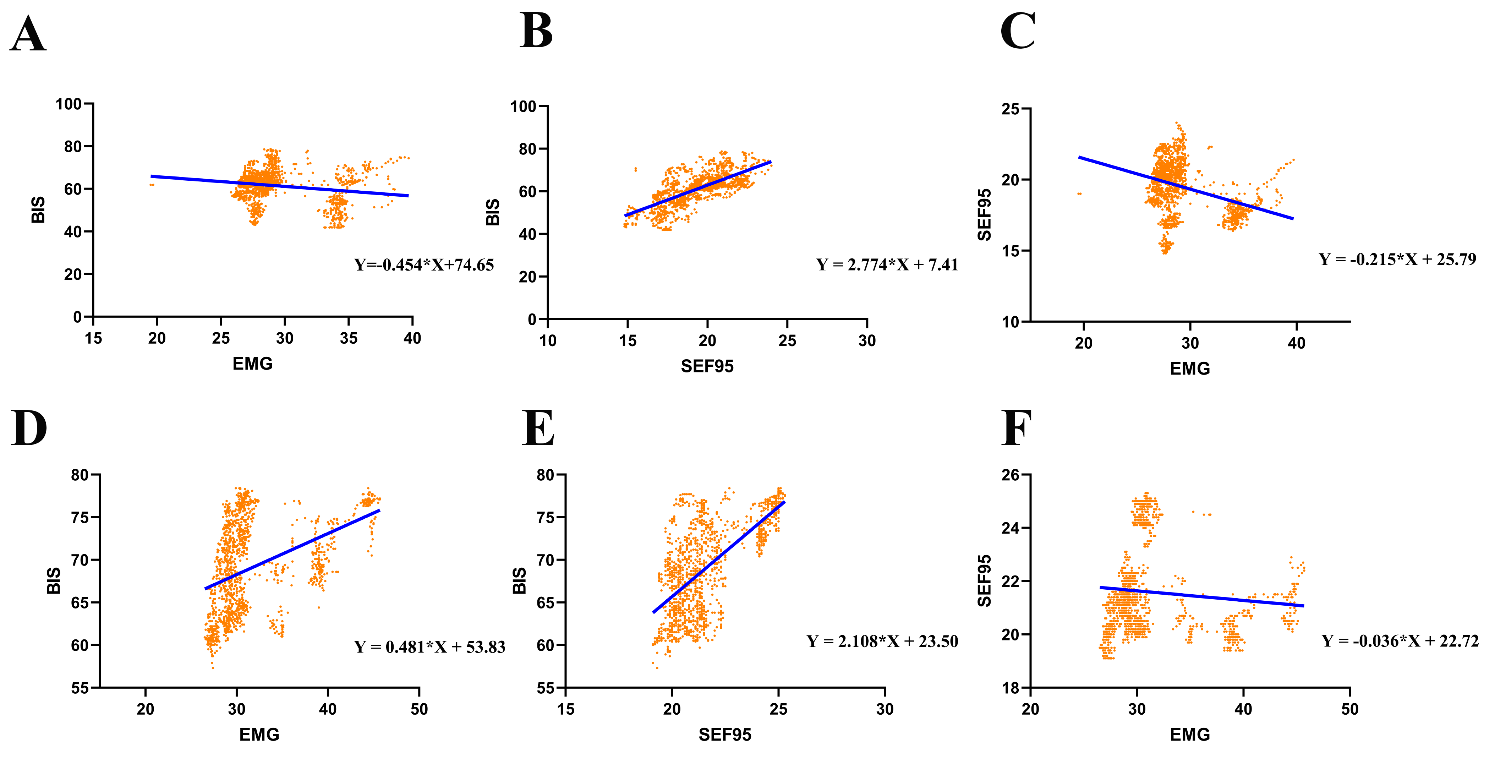


**Supplementary Figure 2.** Linear regression for A: BIS and EMG, B: BIS and SEF95, and C: SEF95 and EMG under propofol anesthesia in females; Linear regression for D: BIS and EMG, E: BIS and SEF95, and F: SEF95 and EMG under remimazolam anesthesia in females.

## Supplementary Tables

**Table 1** **Comparison of BIS, EMG, and SEF95 between the P group and the RM group in males**

|  | P Group (n=8) | RM Group (n=9) | Cohen's d | *p* values |
| --- | --- | --- | --- | --- |
| BIS | 58.40 ± 6.86 | 70.22 ± 6.73 | 1.743 | *p*<0.001 |
| EMG (dB) | 29.72 ± 2.60 | 34.64 ± 5.91 | 1.057 | *p*<0.001 |
| SEF95 | 17.93 ± 2.52 | 21.21 ± 1.74 | 1.531 | *p*<0.001 |

**Note:** Data are presented as mean ±SD and number

**Abbreviations:** P, propofol; RM, remimazolam; BIS, bispectral index; EMG, electromyogram; SEF95, 95% spectral edge frequency.

**Table 2** **Comparison between EMG and BIS vs. EMG and SEF95** **under propofol anesthesia in males**

|  | Spearman’s correlation coefficient and 95% CI | Linear model | 95% CI of the slope | F-statistics | *p* values | R^2^ |
| --- | --- | --- | --- | --- | --- | --- |
| BIS | -0.237 [-0.275, -0.199] | BIS=-0.625*EMG+ 76.97 | [-0.728, -0.523] | 143.1 | *p*<0.0001 | 0.056 |
| SEF95 | -0.481 [-0.512, -0.450] | SEF95=-0.477*EMG+32.10 | [-0.511, -0.443] | 770.8 | *p*<0.0001 | 0.243 |

**Note:** Data are presented as number, 95%confidence interval, and number (95% confidence interval)

**Abbreviations:** BIS, bispectral index; CI, confidence interval; EMG, electromyogram; SEF95, 95% spectral edge frequency.

**Table 3 Comparison between EMG and BIS vs. EMG and SEF95 under remimazolam anesthesia in males**

|  | Spearman’s correlation coefficient and 95% CI | Linear model | 95% CI of the slope | F-statistics | *p* values | R^2^ |
| --- | --- | --- | --- | --- | --- | --- |
| BIS | 0.374 [0.341, 0.406] | BIS=0.480*EMG+53.61 | [0.441, 0.519] | 582.8 | *p*<0.0001 | 0.178 |
| SEF95 | 0.168 [0.131, 0.204] | SEF95=0.042*EMG+19.74 | [0.031, 0.053] | 57.0 | *p*<0.0001 | 0.021 |

**Note:** Data are presented as number, 95% confidence interval, and number (95% confidence interval)

**Abbreviations:** BIS, bispectral index; CI, confidence interval; EMG, electromyogram; SEF95, 95% spectral edge frequency.

**Table 4** **Comparison between SEF95 and BIS under propofol and remimazolam anesthesia in males**

|  | Spearman’s correlation coefficient and 95% CI | Linear model | 95% CI of the slope | F-statistics | *p* values | R^2^ |
| --- | --- | --- | --- | --- | --- | --- |
| BIS (P group) | 0.807 [0.797, 0.816] | BIS=1.942*SEF95+23.58 | [1.866, 2.019] | 2477 | *p*<0.0001 | 0.508 |
| BIS (RM group) | 0.543 [0.516, 0.569] | BIS=2.389*SEF95+19.56 | [2.274, 2.503] | 1672 | *p*<0.0001 | 0.383 |

**Note:** Data are presented as number, 95% confidence interval, and number (95% confidence interval)

**Abbreviations:** P, propofol; RM, remimazolam; BIS, bispectral index; CI, confidence interval; SEF95, 95% spectral edge frequency.

**Table 5** **Comparison of BIS, EMG, and SEF95 between the P group and the RM group in females**

|  | P Group (n=6) | RM Group (n=5) | Cohen's d | *p* values |
| --- | --- | --- | --- | --- |
| BIS | 61.53 ± 6.85 | 69.01 ± 5.38 | 1.215 | *p*<0.001 |
| EMG (dB) | 29.19 ± 2.82 | 31.52 ± 4.60 | 0.612 | *p*<0.001 |
| SEF95 | 19.51 ± 1.77 | 21.58 ± 1.65 | 1.213 | *p*<0.001 |

**Note:** Data are presented as mean ±SD and number

**Abbreviations:** P, propofol; RM, remimazolam; BIS, bispectral index; EMG, electromyogram; SEF95, 95% spectral edge frequency.

**Table 6 Comparison between EMG and BIS vs. EMG and SEF95 under propofol anesthesia in females**

|  | Spearman’s correlation coefficient and 95% CI | Linear model | 95% CI of the slope | F-statistics | *p* values | R^2^ |
| --- | --- | --- | --- | --- | --- | --- |
| BIS | 0.050 [0.003, -0.096] | BIS=-0.454*EMG+ 74.65 | [-0.565, -0.344] | 65.0 | *p*<0.0001 | 0.035 |
| SEF95 | -0.198 [-0.242, -0.153] | SEF95=-0.215*EMG+25.79 | [-0.243, -0.188] | 238.7 | *p*<0.0001 | 0.117 |

**Note:** Data are presented as number, 95%confidence interval, and number (95% confidence interval)

**Abbreviations:** BIS, bispectral index; CI, confidence interval; EMG, electromyogram; SEF95, 95% spectral edge frequency.

**Table 7 Comparison between EMG and BIS vs. EMG and SEF95 under remimazolam anesthesia in females**

|  | Spearman’s correlation coefficient and 95% CI | Linear model | 95% CI of the slope | F-statistics | *p* values | R^2^ |
| --- | --- | --- | --- | --- | --- | --- |
| BIS | 0.533 [0.496, 0.569] | BIS=0.481*EMG+53.83 | [0.428, 0.535] | 306.4 | *p*<0.0001 | 0.170 |
| SEF95 | 0.132 [0.082, 0.182] | SEF95=-0.036*EMG+22.72 | [-0.054, -0.018] | 15.25 | *p*<0.0001 | 0.010 |

**Note:** Data are presented as number, 95% confidence interval, and number (95% confidence interval)

**Abbreviations:** BIS, bispectral index; CI, confidence interval; EMG, electromyogram; SEF95, 95% spectral edge frequency.

**Table 8 Comparison between SEF95 and BIS under propofol and remimazolam anesthesia in females**

|  | Spearman’s correlation coefficient and 95% CI | Linear model | 95% CI of the slope | F-statistics | *p* values | R^2^ |
| --- | --- | --- | --- | --- | --- | --- |
| BIS (P group) | 0.733 [0.711, 0.754] | BIS=2.774*SEF95+7.41 | [2.649, 2.899] | 1900 | *p*<0.0001 | 0.514 |
| BIS (RM group) | 0.606[0.573, 0.637] | BIS=2.108*SEF95+23.50 | [1.983, 2.234] | 1078 | *p*<0.0001 | 0.419 |

**Note:** Data are presented as number, 95% confidence interval, and number (95% confidence interval)

**Abbreviations:** P, propofol; RM, remimazolam; BIS, bispectral index; CI, confidence interval; SEF95, 95% spectral edge frequency.
